# Supplementary material for: Behavioural determinants of testing behaviour during a hypothetical human-to-human avian influenza outbreak: an interview study
Source: BMC Public Health. 2026 Jun 25;26:2908. doi: 10.1186/s12889-026-28252-w (PMC13651048; doi:10.1186/s12889-026-28252-w)
Supplement: Supplementary file 2 [file 12889_2026_28252_MOESM1_ESM.docx]

# Additional file 1: Interview guide

**SOCIO-DEMOGRAPHIC INFORMATION**

Age in years, gender, municipality, region of the Public Health Services, highest completed education, occupation, country of origin of the respondent, country of origin of parents, COVID-19 vaccination status

**BEFORE SCENARIO IS PRESENTED**

| SUB-QUESTIONS | STARTING QUESTIONS FOR RESPONDENT |
| --- | --- |
| -What knowledge is present about avian influenza? -What knowledge is present about testing? | -What comes to mind when you think of infectious diseases? -How do you feel about testing for infectious diseases? -What comes to mind when you think of avian influenza? -How do you feel about testing for avian influenza? |

**AFTER INTRODUCING THE ANIMATION**

| SUB-QUESTIONS | STARTING QUESTIONS FOR RESPONDENT |
| --- | --- |
| -How does the perception of testing and avian influenza change after the animation introduction? | -Did you understand everything you just saw? If not, would you like to see the video again? -What do you think when you see this? -How do you now feel about testing for avian influenza? |

**AFTER PART 1 OF THE ANIMATION**

| SUB-QUESTIONS | STARTING QUESTIONS FOR RESPONDENT |
| --- | --- |
| -Control question: does the respondent understand the animation? | -Did you understand everything you just saw? If not, would you like to see the video again? |
| -What is the perceived risk regarding avian influenza? -What are the intentions towards testing? | -What do you think when you see this? -Are you worried or not worried about what you saw? -Would you get tested after seeing this video? Why or why not? -Can you imagine situations where you would (or would not) do it? |

**AFTER PART 2 OF THE ANIMATION**

| SUB-QUESTIONS | STARTING QUESTIONS FOR RESPONDENT |
| --- | --- |
| -Control question: does the respondent understand the animation? | -Did you understand everything you just saw? If not, would you like to see the video again? |
| -What is the risk perception regarding avian influenza? -What are the intentions regarding testing? | -What do you think when you see this second part? -Would you still get tested or not, and for what reasons? -Why now yes/no? -Can you imagine situations where you would (or would not) do it? |

**FOLLOW-UP**

| SUB-QUESTIONS | STARTING QUESTIONS FOR RESPONDENT |
| --- | --- |
| What knowledge/risk perception exists regarding the disease? | After watching the video: what would you like to know more about regarding avian influenza and symptoms? What do you think of the possible symptoms and consequences? Why is testing done? Are these also reasons for you to test? |
| What are the ideas about the possible consequences of testing or not testing? | What do you think are the possible consequences of testing or not testing? What are your personal consequences? And for society? Are the possible consequences of not testing important to you? |
| Which health literacy skills are present? Ability to book a test, follow self-test procedure, interpret results? | How would you obtain information about testing or avian influenza? Was it clear in the past how to book a test appointment? Were there barriers? How did you book appointments (phone/computer)? How do you experience doing a self-test? How do you interpret the result? |
| Are there any previous testing experiences? | Have you tested before? Tell more. Why did you previously test or not test? |
| Which emotions are underlying the idea of being tested? | How do you feel about the idea of being tested? What emotions does it evoke? Do you think you might regret not testing? |
| To what extent do social pressure / group norms are underlying testing behaviour? | How do people around you feel about testing? Do you care what others think? How do you feel about online/social media/news messages? |
| Do social/professional roles are underlying testing behaviour? | Would you tell family/friends you tested? Why yes/no? Is this different at work? Have you posted about testing on social media? |
| Is there trust in authorities and care providers? Trust in test/results? | How much trust do you have in authorities regarding avian influenza information? What if information comes from healthcare providers? Do you trust test results done by others? Do you trust self-test results? |
| To what extent do personal goals/motives play a role? | What are reasons for you to test or not test? How do factors like own health, others, work, activities play a role? What is the most important reason for you to test? |
| To what extent does access (distance, cost, opening times) is underlying willingness? | Why would you go/not go to a test location? How do waiting times influence your decision? What do you think of test costs? |
| Which environmental factors facilitated or hindered testing during COVID-19, and how does this relate to avian influenza? | Do things like financial incentives or access influence testing? What influenced you earlier (COVID)? Did campaigns or signage influence you? Did positive/negative results change behaviour? How would this influence avian influenza? Does type of test matter? |
