# Supplementary material for: Behavioural determinants of testing behaviour during a hypothetical human-to-human avian influenza outbreak: an interview study
Source: BMC Public Health. 2026 Jun 25;26:2908. doi: 10.1186/s12889-026-28252-w (PMC13651048; doi:10.1186/s12889-026-28252-w)
Supplement: Supplementary file 3 [file 12889_2026_28252_MOESM2_ESM.docx]

# Additional file 3

Tabel S1. Demographic information reported by the participants

| Pseudonym | Gender | Age | Country of birth | Country of birth parents | Education level | Willingness to test | Perceived autonomy and freedom of choice | Trust in governmental authorities |
| --- | --- | --- | --- | --- | --- | --- | --- | --- |
| 1 | Female | 70+ | Netherlands | Netherlands | Higher | Higher | More general: everyone can make their own choice | Higher |
| 2 | Male | 60-69 | Netherlands | Netherlands | Higher | Lower | Strong need for autonomy/freedom of choice | Lower |
| 3 | Female | 18-28 | Netherlands | Netherlands | Lower | Higher | Not mentioned | Higher |
| 4 | Male | 18-28 | Netherlands | Turkey | Moderate | Lower | Everyone can make their own choice | Lower |
| 5 | Female | 60-69 | Netherlands | Netherlands and Germany | Moderate | Higher | Not mentioned | Higher |
| 6 | Female | 29-39 | Netherlands | Netherlands | Lower | Lower | Strong need for autonomy/freedom of choice | Lower |
| 7 | Female | 50-59 | Netherlands | Netherlands | Moderate | Lower | Strong need for autonomy/freedom of choice | Unclear |
| 8 | Male | 40-49 | Suriname | Suriname | Moderate | Higher | Not mentioned | Higher |
| 9 | Female | 50-59 | Netherlands | Netherlands | Higher | Lower | Strong need for autonomy/freedom of choice | Lower |
| 10 | Female | 60-69 | Netherlands | Netherlands | Lower | Lower | Need for autonomy/freedom of choice | Lower |
| 11 | Female | 40-49 | Netherlands | Netherlands | Higher | Lower | Strong need for autonomy/freedom of choice | Lower |
| 12 | Female | 29-39 | Netherlands | Netherlands | Higher | Lower | Strong need for autonomy/freedom of choice | Lower |
| 13 | Male | 29-39 | Netherlands | Netherlands | Lower | Lower | Strong need for autonomy/freedom of choice | Higher |
| 14 | Female | 29-39 | Suriname | Suriname | Higher | Higher | Everyone can make their own choice | Higher |
| 15 | Male | 50-59 | Suriname | Suriname | Lower | Higher | Everyone can make their own choice | Higher |
| 16 | Vrouw | 40-49 | Netherlands | Turkey | Lower | Higher | Not mentioned | Unclear |
| 17 | Vrouw | 40-49 | Iraq | Iraq | Lower | Lower | Need for autonomy/freedom of choice | Unclear |
